# The Role of Eye Movement Driven Attention in Functional Strabismic Amblyopia

**DOI:** 10.1155/2015/534719

**Published:** 2015-03-09

**Authors:** Hao Wang, Sheila Gillard Crewther, Zheng Qin Yin

**Affiliations:** ^1^Southwest Eye Hospital/Southwest Hospital, Key Laboratory of Visual Damage and Regeneration and Restoration of Chongqing, Third Military Medical University, Chongqing 400038, China; ^2^School of Psychological Science, Faculty of Science, Technology and Engineering, La Trobe University, Melbourne, VIC 3086, Australia

## Abstract

Strabismic amblyopia “blunt vision” is a developmental anomaly that affects binocular vision and results in lowered visual acuity. Strabismus is a term for a misalignment of the visual axes and is usually characterized by impaired ability of the strabismic eye to take up fixation. Such impaired fixation is usually a function of the temporally and spatially impaired binocular eye movements that normally underlie binocular shifts in visual attention. In this review, we discuss how abnormal eye movement function in children with misaligned eyes influences the development of normal binocular visual attention and results in deficits in visual function such as depth perception. We also discuss how eye movement function deficits in adult amblyopia patients can also lead to other abnormalities in visual perception. Finally, we examine how the nonamblyopic eye of an amblyope is also affected in strabismic amblyopia.

## 1. Introduction 

Amblyopia is traditionally defined as reduced vision in one eye due to early, nonsynchronous binocular inputs during the “critical period” (CPs) of visual plasticity in childhood (see [1] for extensive review). This is typically caused by misalignment of visual axes (strabismus), visual deprivation, and/or unequal refractions in the two eyes (anisometropia) and acquired esotropia. Amblyopia resulting from strabismus usually prevents normal binocular fixation and synergy of binocular eye movement functions [2].

Eye movements to a particular object in space necessarily shift attention to that place. Indeed, humans and animals cannot move their eyes to a particular location if their attention is fixed on a different place or object [3]. Attention can be shifted overtly by rapid eye movements [4], such as saccades, or covertly without any movement of the eyes but this function does not develop until late childhood. Research in both humans and monkeys has shown that stable fixation helps the visual system enhance visual attention [5]. This is important behaviourally as visual attention plays a key role in visual perception, behavioral guidance, learning, and short-term memory and working memory [6]. Thus, eye movement skills and ability to fixate stably are critical factors for normal visual attention though often seriously impaired [7] in strabismic amblyopes.

The allocation of attention in adults is usually controlled by the joint interaction of conscious top-down goal directed attention and incoming visual stimuli driving involuntary bottom-up mechanisms [4]. Usually the top-down attention dominates behavior but if the incoming environmental information relates to unexpected motion it will “grab attention” as moving stimuli have the potential to be evolutionarily dangerous and attention may be required to facilitate rapid assessment of the direction and source of the movement and whether a rapid responsive action is required. If not life-threatening or salient, for example, another child waving their hand unthreateningly in the environment, the individual is likely to veto the information and return attention to the previous object of interest. Such conservation and allocation of attentional resources is thus essential for development of normal visual function in childhood and normal visual perception in adulthood. Thus, we suggest that abnormal attention function due to eye movement dysfunction is a significant consequence of strabismic amblyopia. This leaves us with an important unanswered question: is the visual attention dysfunction resulting from abnormal eye movement skills a further underlying cause of visual function deficits in the amblyopic eye?

## 2. The Neural Networks of Visual Attention

The adult human cortical visual system is broadly organized into two segregated pathways in the posterior half of the brain, the dorsal and ventral streams [8, 9]. The* dorsal (where) stream* is primarily driven by the faster conducting magnocellular (M) pathway that projects from retina to thalamus to visual areas V1 and/or V5 then to posterior parietal cortex (PPC) and is primarily concerned with spatial orientation, location of stimuli, activation of selective attention, and visually driven actions [10]. The* ventral (what) stream* receives joint input from both the retinally derived M and the slower parvocellular (P) projections to inferotemporal cortex and object recognition.

When considering the neural networks associated with goal directed attention, Corbetta and colleagues [11] have used functional magnetic resonance imaging to further divide the faster dorsal visual stream into a more dorsal network, extending from the intraparietal cortex to the superior frontal cortex and including frontal eye fields (FEF) that is involved in preparing and applying voluntary goal directed (top-down) eye movement driven selection of stimuli and action. The second attention network in dorsal cortex includes the temporoparietal cortex and inferior frontal cortex, is largely lateralized to the right hemisphere, and is specialized for the involuntary detection of behaviourally relevant incoming “bottom-up” stimuli, particularly when they are salient or unexpected. Neural activation in the SPL and FEF can also influence neural activities in the earlier visual cortex and in the subcortical system [12, 13]. Indeed, the timing of activities between the PPC and earlier visual areas (the primary visual cortex and the medial temporal area) becomes synchronized when macaques selectively attend to a location [14].

The idea of attention as a time and space limited resource followed much debate in the late 1980s and 1990s about attention as a bottleneck for cognitive processing. To account for these limitations on object recognition and attention, Treisman proposed her Feature Integration Theory of visual processing with the idea that there could only be one “spotlight of attention”; that is, attention can only be given to one object at a time [15]. Research performed by Reynolds et al. on monkeys has supported this idea [16]. Modulation of attention can change with both neuronal tuning and stimulus contrast [17, 18]. Reynolds et al. also noted that the neuronal firing rate in the V4 area increases with rise in stimulus contrast in a nonattention situation [16]. Attention also enhances the neuronal firing rate in the same stimulus contrast but cannot enhance the highest firing rate. Other studies have shown that attention can enhance the gain of neural responses at the same stimulus contrast, even at the highest contrast of stimulus [19, 20]. This kind of difference may be due to the size relationship between stimulus and attention window [21]. When the size of the stimulus is smaller than the attention window, the former phenomenon can be observed; in contrast, when the stimulus is larger than the attention window, a gain of neural response is seen. Attention also reduces interneuronal correlations [22]. Specifically, it reduces correlated noise in the visual processing system and improves information processing, thus making neural processing more effective and accurate.

As described above, attention can usually be considered the bottle neck of information processing; however, if there is unexpected motion in the visual environment, the bottom-up stimulus will “grab” attention and shift eye fixation away from an object under top-down consideration [10] to the moving object. Bottom-up attention is necessarily grabbed by the motion-selective hMT+/V5 complex. Attention is also influenced by nonspatial features, such as color, shape, spatial frequency, motion, and orientation [23, 24], via the frontoparietal networks and color-selective area V4/V8. hMT+/V5 can also be activated by attending to color or motion features, even in the absence of related visual stimulation [24–26]. Thus, the object of attention and attention allocation is selective and unitary in time and space; in this manner, distraction is avoided though not as well through the amblyopic eye.

## 3. Visual Attention in Childhood

Early in life, the allocation of attention is involuntarily controlled by the unexpected bottom-up input in the environment. However, as the allocation of attention develops with age, the child becomes progressively more goal directed and attention is more voluntarily controlled. Voluntary decisions utilize previous experience of what is important and/or likely to be evolutionarily salient for decision making for action. Thus, stored visual experience drives behavior (action) via the interaction of the top-down attention system that is goal directed and facilitates the rapid eye movements required to find the appropriate incoming image of a sought object. Furthermore, adequate development of control of fixational eye movements and hence development of attention is essential for the development of normal visual function in childhood and normal visual perception in adulthood. Thus, we suggest that impaired ability to fixate binocularly due to the slower nonattention driving eye movements of the strabismic eye is a significant consequence of strabismic amblyopia.

As indicated above, there are several neural networks subserving the different attentional functions of rapid (transient attention driven primarily by the M-subcortical pathway) selective attention, sustained detailed ventral stream driven attention, and the more executive control networks of the frontal cortex [27, 28]. The development of these three subsystems is not uniform; they have different onsets and rates of development [29, 30] with motion driven magnocellular function coming on line first, but taking till late adolescence to finally mature while the P pathways that subserve high spatial frequency and high acuity vision reache maturity by 6 years of age [31, 32]. However, it is the high acuity P system which is compromised in strabismic amblyopia in animal models [33]. During the first month of life, infants can selectively shift attention from one fixation target to another, such as a moving object in the periphery of their visual field [34]. However, this ability is easily disrupted in one-month-old infants because it only involves the subcortical system, that is, the superior colliculus (SC) [35], as the synaptic development in the visual cortical system underlying binocular vision is usually not considered to be functional till 12–16 weeks [36]. By three months of age, synaptic development in the visual cortical system including connections in different cortex areas and connections between cortex areas and subcortical structures, such as the SC, is proceeding rapidly. As a result, infants' fixation and shifting behaviors become more stable and their binocular functions begin to emerge [34, 36] so that many three-month-olds can follow objects more smoothly and fixate on moving targets more accurately than younger infants [37, 38]. At approximately six months of age, shifts in attention begin to be affected by preceding stimuli [39], and these relate to a covert process of spatial attention, which gives rise to a subsequent saccade [40]. Even at 8 to 12 months, infants are still distracted by former familiar stimuli [41] though by then many are beginning to show an increasing ability to sustain their attention goal [42]. However, sustained attention is still immature compared to that of adults presumably because prefrontal cortex function, which is related to executive control of attention, develops slowly in the human brain from childhood to young adulthood [43, 44].

Between 1 and 3 years of age, the prefrontal cortex gradually develops. Both sustained attention and familiar tendency inhibition become more developed. In attention competition tasks, toddlers can avoid fixating on distraction. Furthermore, the latency time of shifting attention becomes shorter, and ocular motor behaviors are better controlled [27, 41]. By 3–6 years of age, children acquire the ability to fixate stably and sustain attention on a particular object by following with a sequential series of organized saccades. However, their attention can vary with the demands and their interest in those demands [45]. Nonetheless, their executive attention ability is still less than that in older children and adults [46]. Additionally, it can be constrained by working memory, basic neural processing speed, and other limitations [47–49]. From 6 years of age until adolescence, the speed of prefrontal cortex development declines and is ultimately completed [50]. Following this, overall cortical function matures into adulthood. Brain MRI studies show that gray matter volume generally decreases during this period. In contrast, white matter volume linearly increases [51]. Cortex thickness in the frontal, parietal, and occipital lobes (all associated with visual function) decreases, while it increases in brain regions associated with language function [52]. This demonstrates that the dramatic development of cortical connections is essential for advanced visual functions, including attention [44].

## 4. How Do Eye Movement Deficits Result in Abnormal Visual Development in Childhood?

The specific relationship between abnormal eye movement function deficits in children and their visual function development is largely unknown. Normal visual system development requires adequate visual stimulation in the CPs of visual cortex plasticity [53]. The peak of CPs plasticity in humans is 3-4 years after birth; this plasticity ends by 7–10 years of age [54]. At the start of visual system development, the subcortical system establishes connections with the visual cortex. Once the visual cortex receives enough binocular visual input (~12 weeks), it begins to develop and enter the CP. Abnormal eye movements and asynchronous fixation of the strabismic eye would disrupt binocular visual cortex development at its earliest stage [1]. Additionally, it would impair the ability of the subcortical system to connect with the visual cortex and, thus, limit its development.

Involuntary eyeball micromovements, such as tremor (physiological nystagmus) [55], drift [56], and microsaccades [57], are important for normal visual function [58]. Such movements help prevent retinal fading and adaption [59, 60]. Research performed in primates noted that these involuntary micromovements could enhance the sensitivity of visual system neurons [61–63] and the level of visual attention [64, 65]. However, accurate visual perception requires stable fixation. Thus, there is a subtle contradictory balance between eye micromovements and fixation. Steady central fixation is an essential requirement for binocular visual attention. Attention can also inhibit the magnitude of eyeball saccades and peripheral perception [5]. In strabismic amblyopia, excessive eye drift, unsteady fixation, and saccadic intrusions can disrupt the balance [1]. As a result, visual attention and perception function deficits of the deviated eye may occur.

The superior colliculus (SC) is the first control center of eyeball micromovements. The circuits of saccade-related burst neurons in the SC result in microsaccades [66, 67]. Tremors and drifts are likely due to neural noise and variable neuronal firings, which influence ocular muscles [68]. In the earliest stages after birth, the strabismic eye by virtue of its different alignment cannot receive stable and effective binocular visual stimulation and hence fixation is driven by the nondeviating eye. The bottom-up stimuli system through the strabismic eye is unlikely to be as effective in grabbing and driving attention. Strabismus also generates more abnormal neural circuits and noise signals in the subcortical system and influences the function of selective attention [69]. After several months, the development of connections between the subcortical system and the cortical system (occipital, parietal, and frontal cortex) is also disrupted. Therefore, the binocular functions of the top-down control system also show impairments and the development of sustained attention function becomes progressively more limited.

Cortical functions are enhanced as the visual system develops. As indicated above, research in both humans and monkeys shows that the lateral intraparietal area (LIP) of the parietal cortex and the frontal eye field (FEF) of the prefrontal cortex are the specific regions involved in the allocation of attention [4, 70, 71]. These areas also guide eye movement functions; thus, they are associated with overt attention shift [72]. Thus, unstable binocular fixation through the strabismic eye affects binocular eye movement functions and further exacerbates abnormal functional development of the brain through the misaligned eye. These functional deficits also affect the binocular driving of visual attention and associated executive functions. All of these effects result in abnormal development of attentional functions during the CP of development of the visual system and eventually lead to impaired acuity through the strabismic eye and functional amblyopia [73]. Asynchronous abnormal visual input influences the development of the primary visual cortex (V1) and later visual processing streams. Asynchronous input through the two eyes also results in poor contrast sensitivity, global shape perception, and temporal processing of the amblyopic eye. Since visual attention is mostly driven by the nonamblyopic eye at all stages of development, there is suppression that often develops between the nonamblyopic eye and the amblyopic eye in adulthood.

## 5. Visual Attention in Adult Visual Perception

Most of the visual information that falls on an eye is ignored by the visual system. For example, when driving a car or riding a bicycle one does not remember every vehicle or person that passes on the other side of the road. Such information is irrelevant to the primary goal directed purpose of driving somewhere safely. Prior learning of how to drive a vehicle safely and experience play an important role in all top-down behaviors, such that the moment the driver sits in the driving seat his/her brain anticipates all the potential information that is likely to be relevant for efficient travelling and safety. Hence, particular aspects of the visual environment are expected, for example, traffic lights and cars turning out of corners, and processed for action and sometimes encoded in memory while objects on the footpath nearby are usually filtered out and not remembered; that is, such information is unnecessary and irrelevant and is potentially a distractor taking up time and important neural resources [74]. Eye movements play an essential role in visual perception and are also the basis of visual attention. As mentioned previously, eyeball micromovements help maintain the retinal image and hence our perception of stationary and moving objects [75]. Moving unexpected stimuli in the environment are highly likely to involuntarily “grab” our visual attention as such moving objects are potentially dangerous and evolutionarily important. Such bottom-up information will grab attention and feed forward very rapidly to superior colliculus and frontal eye fields and to parietofrontal cortical areas to influence consciousness and executive decisions to sustain attention and spatial shifting of the eyes under the direction of top-down feedback signals [76, 77]. Each of these adjusts our attention allocation by controlling eye saccade movements, including saccade latency and saccade trajectory [78, 79]. When an object is viewed in the presence of distractors, the stimulus-driven (bottom-up) signals lead to shorter saccade latency [80, 81]. Next, eye movements are adjusted by both goal-driven (top-down) and bottom-up signals. Such movements can suppress irrelevant oculomotor activities or increase saccade latency, to ensure adequate processing [82].

## 6. How Do Eye Movement Driven Attention Deficits Result in Abnormal Visual Perception in Adult Strabismic Amblyopia?

Visual perception deficits in adult strabismic amblyopia cannot be cured by current therapies, presumably because the underlying cause is abnormal development of the visual system in childhood and the fact that plasticity for rehabilitation of the visual cortex declines later in development after the end of the early CP of development of the visual cortex [83]. Deficiencies associated with amblyopia include low visual acuity, reduced contrast sensitivity, abnormal contour interaction (crowding), visual stimuli space localization disability, oculomotor function deficits, spatial and temporal information processing deficits, and some higher level function limitations (undercounting and missing problems) [84–89]. Abnormal attention functions due to impaired binocular eye movement skills and poor fixation functions in adults would also influence visual perception [90].

The abnormal development results in many visual neural processing problems in the adult amblyope. In humans, the functioning of the neural networks in the brain associated with the amblyopic eye is different than that driven by the nonamblyopic eye [91]. Functional imaging research has shown that the ocular dominance columns in V1 of an amblyopic eye are dramatically decreased, and this cannot be altered by therapy in the adult [92, 93]. Asynchrony of incoming monocular visual information also limits the binocular connections in V1 but does not totally suppress the number of neurons driven by the amblyopic eye [85]. On the other hand visual acuity in V1 is dominated by the nondeviated eye. Indeed, the acuity of the amblyopic eye is suppressed in cats, monkeys, and humans [94–97]. Thus, the bottom-up system of the amblyopic eye, which includes dorsal and ventral stream processing, is also suppressed by object related features but not by unexpected movement [1].

Vision through the strabismic eye disrupts spatial- and object-based attention. The lowered acuity of the amblyopic eye makes it difficult to rapidly make a feature discrimination between an object and a distraction (e.g., an e or a c letter); as a result, the strabismic eye is less able to dominate attention and direct eye movements and hence fixation on an object becomes progressively slower and more impaired. This idea is supported by research in humans showing that activation of cortical networks dramatically decreases in the amblyopic eye [98]; effective connectivity is also lost in the amblyopes' cortex. The more severe the amblyopia, the more severe the loss [99]. Both dorsal and ventral stream connections are decreased. Both feedforward and feedback connections are equally affected. Strabimic amblyopia also reflects the ability of neurons in different brain areas to fire synchronously [100]. This results in lack of stereopsis and impaired depth perception [1], as well as impaired top-down attention executive control. The prefrontal and posterior parietal areas cannot connect and control the synchronous firing rate of neurons in lower level visual areas. Thus, attention control and allocation functions are limited. Weaker executive attention functions also result in less sustained and goal-driven attention and also influence visual perception through the strabismic eye presumably contributing to central scotomas under binocular viewing conditions.

The abnormal eye movement functions of the amblyopic eye also influence the visual attention and perception function in adults [101]. Unbalanced neural input to eye movement muscles results in eyeball deviations and also causes image focus on a nonfoveal region [1]. This is likely to be another factor contributing to eccentric fixation and the influence on visual attention functions [1, 102]. Improper retinal fixation disrupts the normal fixation reflex and causes poor spatial localization and visually driven motor responses. It also influences the space-based bottom-up attention function and normal visual perception. Poor fixational eye movement skills also result in the crowding effect [103]. The excessive eye drifts and saccades disrupt the normal fixation and visual attention functions and cause low visual acuity of the amblyopic eye. This low visual acuity leads to the contour interaction and also is one of the reasons for the crowding effect [1, 104].

## 7. The Nonamblyopic Eye of an Amblyope Is Also Affected in Strabismic Amblyopia

Suboptimal binocular input during a developmental critical period may also influence the development of the nonamblyopic eye. Much research has shown that the visual function of the nonamblyopic eye is not normal in strabismic children. Although the nonamblyopic eye has normal acuity, contrast sensitivity functions (CSFs) in both the amblyopic and the nonamblyopic eyes are reduced [105]. The visual evoked potential of nonamblyopic eyes in children with amblyopia also shows different visual information processes; thus, the nonamblyopic eye in these children is not normal [106]. The visual decision response times of the amblyope are delayed in both the amblyopic and the nonamblyopic eyes [107].

The absence of binocular visual input impacts the development of cortical connections to downstream areas of the brain, such as the parietal and frontal cortex. Normally, binocular summation can help to increase the visual system signal-to-noise ratio [108] and is a critical factor in visual system maturation. In the amblyopic eye, functional connections between the visual cortex and the frontal cortex may be degraded due to nonuse in a developmentally critical period. Additionally, in the nonamblyopic eye, these connections are also reduced due to absence of fully binocular input and low signal-to-noise ratio in the visual system. The decreased connection between the striate cortex and the extrastriate cortex during the CP would be expected to result in abnormal development of higher-level visual processing, such as binocular fixation and binocular visual attention [109–111].

In the adult amblyope, nonamblyopic eye visual function is also affected, although to a lesser degree than the amblyopic eye. Simmers et al. found that global motion processing in adult human amblyopia is abnormal in both amblyopic and nonamblyopic eyes [112]. The ability of global orientation discrimination and blur-discrimination is decreased in both the amblyopic and the fellow eye [113, 114]. The deficits in the nonamblyopic eye are possibly due to the function of the visual pathway at points where a majority of the neurons are binocular (e.g., extrastriate cortex) or are impaired, for example, in the dorsal pathway. This kind of imbalanced interaction in the extrastriate cortex visual system would reduce the signal-to-noise ratio and disrupt the spatial processing and motion processing function in the dorsal pathway [115].

## 8. Conclusions

Abnormal eye movement skills in strabismus can dramatically influence the development of brain-related functional areas and connections between the subcortical and cortical systems during the early stages of life. Thus, the developments of both binocular and monocular visual attention functions are disrupted. Furthermore, the strabismic eye is even more impaired and unable to drive mature eye movements or execute skilled visual attention functions resulting in abnormal visual perception that can be clinically identified as loss of acuity and strabismic amblyopia in childhood and adulthood. Importantly, the nonamblyopic eye development and visual function are also impaired in amblyopia.
